# Determinants of Bone Mineral Density in Iranian Women with Polycystic Ovary Syndrome

**DOI:** 10.5812/ijem-137594

**Published:** 2023-11-06

**Authors:** Fariba Karimi, Parisa Mardani

**Affiliations:** 1 Endocrinology and Metabolism Research Center, Shiraz University of Medical Sciences, Shiraz, Iran

**Keywords:** Bone Mineral Density, Polycystic Ovary Syndrome, Insulin Resistance, Body Mass Index, Osteocalcin, Phosphate

## Abstract

**Background:**

Whether the endocrine aberrations caused by polycystic ovary syndrome (PCOS) might influence bone density in women of reproductive age is controversial.

**Objectives:**

The present study aimed to compare PCOS women to a control group matched in terms of age and body mass index (BMI) regarding bone indices and to clarify the potential relationship between their hormonal changes and bone density.

**Methods:**

This case-control study consisted of 61 PCOS patients, and 35 women with normal ovulatory function served as controls. Bone parameters, including bone mineral content (BMC) and bone mineral density (BMD) in addition to T- and Z-scores, were measured at the lumbar vertebrae, neck of the left femur, hip, and distal part of the radial bone, using dual-energy X-ray absorptiometry. Blood samples were taken to be tested for biochemical parameters and serum concentrations of insulin, osteocalcin, parathyroid hormone (PTH), vitamin D, follicle-stimulating hormone (FSH), luteinizing hormone (LH), total testosterone, dehydroepiandrosterone sulfate (DHEAS), and estradiol were measured. Insulin resistance was evaluated through the homeostatic model assessment of insulin resistance (HOMA-IR).

**Results:**

The results revealed greater levels of HOMA-IR and total testosterone in PCOS women than in controls. Nevertheless, the two groups were comparable in terms of bone parameters. In the control group, BMI was the only determinant of bone density at most of the skeletal sites. Nonetheless, BMI and HOMA-IR were independently and positively associated with bone indices at the femoral neck (FN) and total hip in the PCOS group. Parathyroid hormone and vitamin D concentrations were not different in the two groups. However, phosphate levels were higher in PCOS patients (P = 0.025). Osteocalcin was inversely correlated to BMI, and both groups had a negative correlation between DHEAS and PTH. Serum phosphate was inversely and independently associated with estrogen in the PCOS group (r = -0.377, P = 0.004).

**Conclusions:**

Body mass index and HOMA-IR were independent and positive determinants of FN and total hip bone density in the PCOS subjects. Nonetheless, in the non-PCOS women, BMI was the only independent determinant of bone density at most of the skeletal sites. Additionally, osteocalcin was inversely correlated with BMI in both groups.

## 1. Background

As a multifactorial disorder, polycystic ovary syndrome (PCOS) is the most common cause of anovulation accompanied by increased androgen levels. Its prevalence is estimated to be 5 - 15% among women of reproductive age (1) and is characterized by oligoovulation or anovulation, clinical or biochemical hyperandrogenism, and polycystic ovaries on ultrasound (1-3). Polycystic ovary syndrome is associated with hormonal and metabolic alterations, including androgen and estrogen disturbances, insulin resistance (IR), and obesity, in addition to low-grade chronic inflammation (2, 3), all of which affect bone health. It has been proposed that androgenic hormones might stimulate the development and maturation of human osteoblasts (4, 5).

Moreover, previous studies have reported that approximately 38 - 88% of PCOS subjects are overweight or obese, which might contribute to the association between IR and PCOS (6, 7). On the other hand, recent studies have introduced hyperinsulinemia as another major metabolic change in PCOS, which might affect bone metabolism through insulin receptors on osteoblasts and secretion of osteocalcin, a bone-derived hormone (8, 9). The current understanding of bone health in women with PCOS is limited due to the complexity of PCOS and the paucity of research in this area. Despite the reported positive effects of obesity, insulin, and androgen excess on bone metabolism, recent studies have obtained controversial results concerning the skeletal impacts of hormonal alterations in women suffering from PCOS (10-12). In addition, PCOS patients have been observed to have a higher incidence of fractures than non-PCOS groups (13). These contradictory results have led to uncertainty about whether PCOS improves or deteriorates bone health.

## 2. Objectives

The current study was designed to determine whether there is a difference between bone density in PCOS and non-PCOS women and if there is a correlation between the existing hormonal profile and bone parameters in these patients.

## 3. Methods

### 3.1. Sample Size Calculation

This study calculated the sample size using NCSS (PASS) software. At the error level of alpha = 0.05 and power = 80% and based on the difference in the mean levels of bone mineral density (BMD) values of the lumbar spine in the two groups and their standard deviation in a previous similar study (12) (1.05 ± 0.12 and 1.21 ± 0.18 in PCOS subjects and controls, respectively), the minimum number of participants in the two groups was determined as 20 subjects in each of them ( n_1_ = n_2_ = [(Z_α/2_ + Z_β_)^2^ × {2(ó)^2^}]/(μ1 - μ2)^2^). However, more subjects were included to apply regression models.

### 3.2. Subjects

This case-control study was conducted on 61 women of reproductive age who suffered from PCOS (mean age: 27.91 ± 7.04 years, range: 18 - 41 years). Polycystic ovary syndrome was diagnosed based on the Rotterdam criteria in the presence of two of the followings: (1) Oligoovulation and/or anovulation (having ≤ 8 periods in a year); (2) biochemical and/or clinical evidence of androgen excess (modified Ferriman-Gallwey (mFG) score ≥ 8 and/or total testosterone ≥ 0.48 ng/mL); and (3) polycystic ovarian morphology identified via ultrasound. The control group consisted of 35 healthy women referred for routine check-ups. They were matched in terms of age and body mass index (BMI) with a mean age of 29.93 ± 5.94 years (age range: 19 - 40 years) and had regular menstrual cycles, defined as menstrual cycles between 21 - 35 days. The participants in the two groups were selected using a consecutive sampling method according to the inclusion and exclusion criteria of the study.

The patients and controls were referred to a chief researcher at the Motahari Clinic of Shiraz University of Medical Sciences, Shiraz, Iran, within 2020 - 2021. Patients with clinical or laboratory evidence of hypercortisolism, elevated prolactin levels, thyrotoxicosis, nonclassic congenital adrenal hyperplasia, and virilizing adrenal cortex or ovary tumors were excluded. It is worth mentioning that none of the patients or controls had received metformin, oral contraceptives, or other medications affecting bones. Additionally, none of the present study’s participants were pregnant or had breastfed within the year prior to the study.

Informed consent was obtained from the participants after explaining the objectives of the study. The participants’ weight and height were measured, and their BMI was calculated by dividing their weight in kilograms by their height in square meters. The waist circumference was measured at the midpoint between the lower border of the rib cage and the iliac crest in the upright position with light clothing using flexible tape. Additionally, hirsutism was evaluated by the mFG score. A single examiner performed the scoring assessment of hirsutism for each patient. A mFG score of 8 or more was considered diagnostic for hirsutism (14). According to the American College of Sports Medicine recommendation, the participants were divided into two groups: Those with less than and those with more than three times as much physical activity a week (15). The participants were asked how many days per week they did any physical activity, including regular walking, cycling, organized sports, physical education classes, and recreational activity. Moreover, blood samples were collected early, between 7 a.m. and 9 a.m., after an overnight fast. The samples were centrifuged immediately, and sera were frozen at -70°C until examination.

The laboratory tests were conducted at the Endocrinology and Metabolism Research Center of Shiraz University of Medical Sciences. The glucose, calcium, phosphorus, and albumin serum levels were measured by enzymatic colorimetric assay using a Dirui autoanalyzer (Dirui, CS-T240, China). The serum concentrations of follicle-stimulating hormone (FSH) (Monobind Inc., Lake Forest, CA926630, USA) and luteinizing hormone (LH) (Monobind Inc., Lake Forest, CA926630, USA), estradiol (Monobind Inc., Lake Forest, CA926630, USA), testosterone (Monobind Inc., Lake Forest, CA926630, USA), dehydroepiandrosterone sulfate (DHEAS) (Monobind Inc., Lake Forest, CA926630, USA), and insulin (Monobind Inc., Lake Forest, CA926630, USA) were measured using enzyme-linked immunosorbent assay (ELISA).

The reference range and sensitivity for FSH, LH, estradiol, testosterone, DHEAS, and insulin were 3 - 12 and 0.134 mIU/mL, 0.5 - 10.5 and 0.054 mIU/mL, 9 - 175 and 8.2 pg/mL, 0.2 - 0.95 and 0.0576 ng/mL, 0.03 - 5.88 and 0.042 μg/mL, and 0.7 - 9 and 0.75μIU/mL, respectively. The serum level of osteocalcin was also measured via ELISA (Immunodiagnostic Systems [IDS], UK). The electrochemiluminescence method measured parathyroid hormone (PTH) concentration using a Cobas E411 (Roche, Germany). The concentration of 25-hydroxy vitamin D was determined by a high-performance liquid chromatography method (Young Lee 9100, South Korea). The reference range and sensitivity for osteocalcin, PTH, and 25 - hydroxy vitamin D were 0.5 - 100 and 0.5 ng/mL, 15 - 65 and 1.2 pg/mL, and 20 - 80 and 0.4 ng/ml, respectively. The homeostatic model assessment of insulin resistance (HOMA-IR) was calculated using the following formula:

HOMA-IR = (fasting plasma glucose [mg/dL] × fasting plasma insulin [μU/mL])/405

Moreover, the Hologic system (A, Horizon W, S/N 300317M, USA) was used to measure BMD, bone mineral content (BMC), T-score, and Z-score in the lumbar vertebrae (LS), neck of the left femur, distal part of the radial bone, and hip using dual-energy X-ray absorptiometry (DXA). Quality control procedures were performed according to the manufacturer’s recommendations to prevent possible baseline drift. The DXA operator’s coefficient of variation (CV) for consecutive measurements was 1%.

### 3.3. Ethics

The Local Ethics Committee of Shiraz University of Medical Sciences approved the study protocol, and the ethics certificate number was IR.SUMS.MED.REC.1399.084.

### 3.4. Statistical Analysis

The mean levels of anthropometric, hormonal, biochemical, and parameters of bone density were compared using the Student’s *t*-test and Mann-Whitney U test in cases of variables with and without normal distribution, respectively. The chi-square test was used to compare the stratified variables. Pearson and Spearman’s correlation tests were employed to analyze correlations between the variables with and without a normal distribution, respectively. Univariate and multiple regression analyses were used to evaluate the impact of possible confounding factors on bone parameters at different skeletal sites. Statistical analyses were performed by SPSS software (version 18; Chicago, IL, USA), and P-values less than 0.05 were considered statistically significant.

## 4. Results

The participants’ anthropometric indices, biochemical measurements, and bone parameters are presented in Table 1. 

**Table 1.** Anthropometric Indices, Biochemical Measurements, and Bone Parameters of Participants in Polycystic Ovary Syndrome and Control Groups ^a^

Variables	PCOS Group	Control Group	P-Value
**Number of participants**	61	35	
**Age (y)**	27.92 ± 7.04	29.93 ± 5.94	0.11 ^b^
**Body weight (kg)**	68.61 ± 12.43	66.58 ± 10.03	0.43 ^b^
**Height (cm)**	158.87 ± 5.38	158.89 ± 6.21	0.71 ^b^
**BMI (kg/m** ^**2**^ **)**	27.24 ± 5.16	26.42 ± 4.02	0.44 ^b^
**WCF (cm)**	82.53 ± 11.00	82.92 ± 10.76	0.89 ^b^
**Physical activity**			0.20 ^c^
< 3 times/wk (n)	22	13	
≥ 3 times/wk (n)	49	12	
**FBS (mg/dL)**	101.15 ± 9.32	104 ± 11.07	0.26 ^d^
**Insulin (µU/mL)**	12.48 ± 6.12	9.64 ± 4.22	0.024 ^d^
**HOMA-IR**	3.22 ± 1.76	2.55 ± 1.20	0.011 ^d^
**Osteocalcin (ng/mL)**	13.83 ± 7.94	10.95 ± 5.38	0.12 ^d^
**Vitamin D (ng/mL)**	24.135 ± 10.520	24.00 ± 9.46	0.70 ^b^
**PTH (pg/mL) **	35.53 ± 21.01	43.34 ± 21.20	0.10 ^b^
**Corrected calcium (mg/dL) **	8.72 ± 0.34	8.63 ± 0.34	0.23 ^b^
**Phosphorus (mg/dL) **	3.99 ± 0.53	3.73 ± 0.49	0.025 ^b^
**Testosterone (ng/mL)**	0.52 ± 0.18	0.35 ± 0.10	0.0001 ^d^
**Estradiol (pg/mL)**	135.04 ± 65.39	127.35 ± 59.03	0.59 ^b^
**DHEAS (µg/mL)**	2.01 ± 0.89	1.63 ± 0.67	0.013 ^d^
**LH (mIU/mL)**	7.90 ± 6.17	4.37 ± 2.82	0.003 ^d^
**FSH (mIU/mL)**	5.52 ± 2.80	5.45 ± 3.03	0.80 ^d^
**LS BMD (g/cm** ^**2**^ **)**	0.99 ± 0.11	1.00 ± 0.12	0.61 ^b^
**LS Z-score**	-0.45 ± 1.02	-0.28 ± 1.13	0.48 ^b^
**FN BMD (g/cm** ^**2**^ **)**	0.80 ± 0.10	0.78 ± 0.12	0.64 ^b^
**FN Z-score**	-0.40 ± 0.98	-0.37 ± 1.09	0.88 ^b^
**TH BMD (g/cm** ^**2**^ **)**	0.92 ± 0.12	0.90 ± 0.14	0.53 ^b^
**TH Z-score**	-0.15 ± 0.98	-0.20 ± 1.16	0.76 ^b^
**DR BMD (g/cm** ^**2**^ **)**	0.56 ± 0.04	0.55 ± 0.04	0.39 ^b^
**DR Z-score**	-0.14 ± 0.75	-0.26 ± 0.76	0.47 ^b^

Abbreviations: PCOS, polycystic ovary syndrome; BMI, body mass index; WCF, waist circumference; FBS, fasting blood sugar; HOMA-IR, homeostatic model assessment of estimated insulin resistance; PTH, parathyroid hormone; DHEAS, dehydroepiandrosterone sulfate; LH, luteinizing hormone; FSH, follicle-stimulating hormone; LS, lumbar spine; FN, femoral neck; TH, total hip; DR, distal radius; BMD, bone mineral density; SD, standard deviation.

^a^ The data are expressed as mean ± SD.

^b^ Independent *t*-test

^c^ Chi-square test

^d^ Mann-Whitney U test

The patients had higher levels of phosphate, LH, testosterone, DHEAS, insulin, and HOMA-IR. However, the two groups were comparable regarding estrogen, osteocalcin, PTH, and vitamin D levels and bone parameters (Table 1). The correlation test results showed no significant correlation between estrogen, PTH, vitamin D, and osteocalcin levels with bone parameters at all skeletal sites in the two study groups. The control group showed an inverse correlation between the testosterone level with femoral neck (FN) BMD and FN Z-score (Table 2). Furthermore, a positive correlation was observed between DHEAS with FN BMD and total hip BMD in the PCOS group (Table 2). Additionally, testosterone and DHEAS levels were not correlated to insulin, HOMA-IR, osteocalcin, or vitamin D values in the two groups. The present study also revealed an inverse correlation of PTH with DHEAS levels in the participants (r = -0.340, P = 0.008 in PCOS subjects; r = -0.438, P = 0.018 in controls).

**Table 2.** Correlation of Bone Parameters with Body Mass Index, Homeostatic Model Assessment of Insulin Resistance, Testosterone, Dehydroepiandrosterone Sulfate, Osteocalcin, and Parathyroid Hormone in Polycystic Ovary Syndrome and Control Groups ^a^

	BMI r(p), No. (%)	HOMA-IR r(p), No. (%)	Testosterone r(p), No. (%)	DHEAS r(p), No. (%)	Osteocalcin r(p), No. (%)	PTH r(p), No. (%)
**LS BMD**						
PCOS group	0.24 ^b^ (0.06)	0.11 ^c^ (0.42)	-0.14 ^c^ (0.27)	0.02 ^c^ (0.87)	-0.18 ^c^ (018)	-0.17 ^b^ (0.20)
Control group	0.30 ^b^ (0.10)	0.04 ^b^ (0.81)	0.01 ^b^ (0.97)	0.23 ^c^ (0.22)	-0.40 ^b^ (0.03)	-0.10 ^b^ (0.61)
**LS Z-score**						
PCOS group	0.25 ^b^ (0.06)	0.10 ^c^ (0.44)	-0.14 ^c^ (0.28)	0.01 ^c^ (0.94)	-0.19 ^c^ (0.15)	-0.16 ^b^ (0.22)
Control group	0.33 ^b^ (0.07)	0.06 ^b^ (0.77)	0.03 ^b^ (0.87)	0.24 ^c^ (0.19)	-0.42 ^b^ (0.02)	-0.11 ^b^ (0.54)
**FN BMD**						
PCOS group	0.53 ^b^ (0.0001)	0.42 ^c^ (0.001)	-0.02 ^c^ (0.88)	0.27 ^c^ (0.03)	0.03 ^c^ (0.83)	-0.09 ^b^ (0.51)
Control group	0.53 ^b^ (0.001)	0.25 ^b^ (0.18)	-0.41 ^b^ (0.02)	0.19 ^c^ (0.32)	-0.16 ^b^ (0.39)	0.33 ^b^ (0.08)
**FN Z-score**						
PCOS group	0.60 ^b^ (0.0001)	0.42 ^c^ (0.001)	-0.04 ^c^ (0.76)	0.22 ^c^ (0.08)	-0.05 ^c^ (0.72)	-0.06 ^b^ (0.66)
Control group	0.63 ^b^ (0.0001)	0.27 ^b^ (0.14)	-0.41 ^b^ (0.03)	0.16 ^c^ (0.38)	-0.19 ^b^ (0.33)	0.32 ^b^ (0.09)
**TH BMD**						
PCOS group	0.61 ^b^ (0.0001)	0.36 ^c^ (0.006)	-0.02 ^c^ (0.88)	0.27 ^c^ (0.03)	-0.14 ^c^ (0.28)	-0.23 ^b^ (0.07)
Control group	0.67 ^c^ (0.0001)	0.37 ^c^ (0.04)	-0.25 ^c^ (0.18)	0.14 ^c^ (0.46)	-0.09 ^c^ (0.63)	-0.13 ^c^ (0.48)
**TH Z-score**						
PCOS group	0.70 ^b^ (0.0001)	0.38 ^c^ (0.003)	-0.02 ^c^ (0.87)	0.20 ^c^ (0.12)	-0.20 ^c^ (0.14)	-0.15 ^b^ (0.24)
Control group	0.71 ^c^ (0.0001)	0.40 ^c^ (0.03)	-0.25 ^c^ (0.19)	0.13 ^c^ (0.48)	-0.11 ^c^ (0.55)	-0.13 ^c^ (0.50)
**DR BMD**						
PCOS group	0.23 ^b^ (0.83)	0.13 ^c^ (0.31)	-0.26 ^c^ (0.05)	0.07 ^c^ (0.57)	0.05 ^c^ (0.71)	-0.06 ^b^ (0.63)
Control group	0.54 ^b^ (0.002)	0.32 ^b^ (0.08)	-0.25 ^b^ (0.18)	-0.14 ^c^ (0.45)	-0.33 ^b^ (0.07)	-0.11 ^b^ (0.55)
**DR Z-score**						
PCOS group	0.20 ^b^ (0.15)	0.09 ^c^ (0.53)	-0.25 ^c^ (0.07)	0.02 ^c^ (0.87)	0.09 ^c^ (0.53)	-0.02 ^b^ (0.87)
Control group	0.59 ^b^ (0.001)	0.33 ^b^ (0.07)	-0.23 ^b^ (0.22)	-0.16 ^c^ (0.41)	-0.35 ^b^ (0.06)	-0.13 ^b^ (0.49)

Abbreviations: PCOS, polycystic ovary syndrome; BMI, body mass index; HOMA-IR, homeostatic model assessment of estimated insulin resistance; DHEAS, dehydroepiandrosterone sulfate; PTH, parathyroid hormone; FN, femoral neck; LS, lumbar spine; TH, total hip; DR, distal radius; BMD, bone mineral density.

^a^ Bivariate correlation

^b^ Pearson correlation coefficient

^c^ Spearman’s correlation coefficient

Body mass index was positively correlated to HOMA-IR in both PCOS subjects (r = 0.447, P = 0.0001) and controls (r = 0.450, P = 0.013). Nonetheless, osteocalcin values were inversely correlated to BMI in two groups (r = -0.474, P = 0.0001 in PCOS patients; r = -0.400, P = 0.032 in controls). Moreover, serum phosphate concentrations were higher in PCOS subjects (P = 0.025). Based on the findings, there was no significant correlation of serum phosphate with testosterone, HOMA-IR, PTH, or vitamin D levels in both groups. Nevertheless, serum phosphate was negatively correlated to estrogen concentrations (r = -0.377, P = 0.004) and positively to osteocalcin levels (r = 0.399, P = 0.002) only in the PCOS group.

On univariate analysis, serum osteocalcin, estrogen, and physical activity of the participants had no significant association with the bone parameters in either skeletal site. However, there was a significant positive association between BMI and HOMA-IR with FN and total hip bone parameters in the PCOS group. However, the control group revealed a positive association between BMI and bone parameters at most of the skeletal sites and between HOMA-IR and total hip bone indices (Table 3). 

**Table 3.** Univariate Linear Regression Analysis for Association of Bone Parameters with Body Mass Index and Homeostatic Model Assessment of Insulin Resistance in Two Groups Separately ^a^

Bone Parameters	PCOS Group		Control Group	
	BMI B ^b^(95% CI) ^c^	HOMA-IR B ^b^(95% CI) ^c^	BMI B ^b^(95% CI) ^c^	HOMA-IR B ^b^(95% CI) ^c^
**LS BMD (g/cm** ^**2**^ **)**	0.005 (0.00 - 0.01)	0.01 (-0.01 - 0.02)	0.01 (-0.00 - 0.02)	0.01 (-0.03 - 0.04)
**LS Z-score**	0.05 (-0.00 - 0.10)	0.07 (-0.08 - 0.23)	0.09 (-0.01 - 0.19)	0.05 (-0.31 - 0.42)
**FN BMD (g/cm** ^**2**^ **)**	0.01 (0.01 - 0.01)	0.02 (0.09 - 0.04)	0.02 (0.01 - 0.03)	0.03 (-0.01 - 0.06)
**FN Z-score**	0.11 (0.07 - 0.15)	0.23 (0.09 - 0.37)	0.17 (0.09 - 0.25)	0.25 (-0.09 - 0.58)
**TH BMD (g/cm** ^**2**^ **)**	0.01 (0.01 - 0.02)	0.02 (0.01 - 0.04)	0.02 (0.01 - 0.03)	0.04 (0.00 - 0.08)
**TH Z-score**	0.13 (0.09 - 0.17)	0.22 (0.77 - 0.36)	0.21 (0.14 - 0.28)	0.36 (0.01 - 0.71)
**DR BMD (g/cm** ^**2**^ **)**	0.002 (0.00 - 0.004)	0.003 (-0.004 - 0.009)	0.005 (0.002 - 0.008)	0.01 (-0.00 - 0.02)
**DR Z-score**	0.03 (-0.01 - 0.07)	0.03 (-0.83 - 0.15)	0.11 (0.05 - 0.17)	0.21 (-0.02 - 0.44)

Abbreviations: PCOS, polycystic ovary syndrome; BMI, body mass index; HOMA-IR homeostatic model assessment of estimated insulin resistance; LS, lumbar spine; FN, femoral neck; TH, total hip; DR, distal radius; BMD, bone mineral density.

^a^ Univariate linear regression analysis

^b^ B, unstandardized Beta

^c^ CI, the confidence interval for Beta

Furthermore, multiple regression analysis revealed a positive and independent association of both BMI and HOMA-IR with FN and total hip bone parameters, even after adjustment for possible confounding factors, including age, testosterone, and DHEAS, in PCOS subjects. Nonetheless, an independent association between BMI and bone indices at most of the studied sites was observed in controls (Table 4). 

**Table 4.** Association of Body Mass Index and Homeostatic Model Assessment of Insulin Resistance with Bone Parameters Adjusted for Age, Testosterone, and Dehydroepiandrosterone Sulfate in Polycystic Ovary Syndrome and Control Groups ^a^

Bone Parameters	PCOS Group		Control Group	
	BMI B ^b^ (95% CI) ^c^	HOMA-IR B ^b^ (95% CI) ^c^	BMI B ^b^ (95% CI) ^c^	HOMA-IR B ^b^ (95% CI) ^c^
**LS BMD**	-0.01 (-0.08 - 0.06)	0.01 (-0.01 - 0.03)	0.01 (0.00 - 0.03)	-0.02 (-0.06 - 0.02)
**LS Z-score**	-0.06 (-0.72 - 0.59)	0.07 (-0.1 - 0.23)	0.11 (-0.004 - 0.234)	-0.14 (-0.51 - 0.22)
**FN BMD**	0.06 (0.01 - 0.12)	0.02 (0.01 - 0.04)	0.02 (0.01 - 0.03)	-0.01 (-0.03 - 0.02)
**FN Z-score**	0.60 (0.08 - 1.13)	0.21 (0.08 - 0.34)	0.19 (0.11 - 0.26)	-0.06 (-0.29 - 0.17)
**TH BMD**	0.08 (0.02 - 0.14)	0.02 (0.01 - 0.04)	0.03 (0.02 - 0.04)	0.00 (-0.03 - 0.03)
**TH Z-score**	0.65 (0.14 - 1.17)	0.18 (0.05 - 0.31)	0.23 (0.15 - 0.31)	-0.01 (-0.23 - 0.24)
**DR BMD**	-0.01 (-0.04 - 0.01)	0.003 (-0.003 - 0.009)	0.005 (0.001 - 0.009)	0.003 (-0.01 - 0.02)
**DR Z-score**	-0.14 (-0.62 - 0.34)	0.05 (-0.07 - 0.16)	0.09 (0.01 - 0.17)	0.06 (-0.18 - 0.30)

Abbreviations: PCOS, polycystic ovary syndrome; BMI, body mass index; HOMA-IR, homeostatic model assessment of estimated insulin resistance; DHEAS, dehydroepiandrosterone sulfate; LS, lumbar spine; FN, femoral neck; TH, total hip; DR, distal radius; BMD, bone mineral density.

^a^ Multivariate regression analysis

^b^ B, unstandardized Beta

^c^ CI, the confidence interval for Beta

Fifty-four women with PCOS had FG scores > 8, and there was no significant association between the hirsutism score and bone parameters. Nonetheless, there was no difference between the oligomenorrheic and non-oligomenorrheic individuals regarding the estrogen level or BMD.

## 5. Discussion

This study examined the possible association between PCOS, bone density, and the existing hormonal profile. The results showed no significant difference between PCOS subjects and controls regarding the means of bone indices at all skeletal sites. However, BMI and HOMA-IR were shown to have an independent, positive association with FN and total hip bone parameters in the PCOS group. Nonetheless, BMI was the only determinant of bone density in the control group. Additionally, osteocalcin level was inversely associated with BMI in the two groups, and PCOS patients had higher serum phosphate levels. To the best of our knowledge, this is the first study on this issue among Iranian women.

Polycystic ovary syndrome is a complex disorder whose various features, such as hyperandrogenemia, obesity, and IR, might affect bone metabolism (16). Although androgen excess is a defining feature of PCOS, research regarding testosterone levels and BMD is sparse and controversial for women. Patients with PCOS have acyclic estradiol production; nevertheless, its concentration is considerably lower than that observed during a normal menstrual cycle (16). It has been emphasized that androgen excess has positive bone effects only in the presence of adequate estrogen concentration (16, 17). Therefore, it was proposed that estrogen depletion manifested through menstrual irregularity and chronic oligo-anovulation might counteract the anabolic impacts of androgens on bones (12). Consistent with this hypothesis, in the present study, the total testosterone level was not an independent predictor of BMD when adjusting for BMI, HOMA-IR, and PCOS status.

In addition, in the current study, there were no differences between the two groups regarding the mean estrogen levels, and no significant correlation was observed between the estrogen levels and bone parameters. The results also revealed no significant differences between the oligomenorrheic and non-oligomenorrheic individuals regarding estrogen concentration and BMD. In this regard, Adami et al. showed that in the subgroup of oligo/amenorrheic PCOS patients, BMD at the spine and FN was comparable to the controls but significantly lower than non-amenorrheic PCOS subjects despite similar estradiol levels (18).

Furthermore, it has been suggested that the relationship between androgens and BMD in individuals with hyperandrogenemia seems more dominant in the bones with a higher percentage of cancellous bone (19). However, the current study findings demonstrated no significant association between both groups’ lumbar (cancellous) bone parameters with DHEAS and testosterone levels. The current study showed a direct relationship between DHEAS and bone indices at FN and total hip in the PCOS group. These contradictory findings might indicate the existence of various thresholds regarding the potency of different androgens for their skeletal influences (4). Moreover, the effects of androgens on bone might be site-dependent (4, 20, 21).

In contrast to the previous studies that mostly evaluated the BMD of either the spine or femur, the present study assessed multiple skeletal sites, including the 1/3 distal radius. However, the current study showed no difference between the groups in terms of bone indices at the distal part of the radial bone.

Furthermore, PCOS might influence bone health through mechanisms not necessarily mediated by gonadal hormones alone. In this context, obesity and IR are commonly encountered in PCOS patients. Although some studies have reported a direct relationship between bone density and obesity, others have shown the opposite and reported an inverse relationship (22). The present study’s results also revealed a positive association between BMI and bone parameters in both groups. However, differences in race and ethnicity might yet be another potential cause of the discrepancies observed among the results of previous studies (23). Therefore, it has been suggested that weight gain in genetically predisposed women to developing PCOS can lead to its clinical and biochemical manifestations. In this regard, the genetic determinants of PCOS have been investigated in recent years (24).

In addition, IR, the other common feature of PCOS, has been suggested to positively impact bone density (25). In this regard, osteocalcin, a bone-derived hormone, is essential to the glucose-bone relationship (26). Several studies have shown that the direct relationship between bone density and insulin level is lost after adjusting for BMI (27). The present study’s results revealed that the positive association of HOMA-IR with FN and total hip bone parameters remained even after adjusting for BMI, age, testosterone, and DHEAS in PCOS patients but not in controls. Nonetheless, recent literature has reported an inverse association between hyperinsulinemia and bone density, especially in younger individuals. Consistent with this idea, reduced levels of osteocalcin, as a bone formation marker, were identified in the presence of IR (28). Therefore, it is possible that local reduction of osteocalcin, as a consequence of IR, could potentially reduce the anabolic action of insulin on bones (28, 29).

Furthermore, the contradictory results of previous studies about the effect of IR on the bone might suggest a threshold for IR in promoting healthy bone. In the current study, the PCOS group had higher levels of insulin and HOMA-IR without correlation to osteocalcin. Still, an inverse relationship was observed between osteocalcin and BMI in both groups. In contrast to the findings of the current study, Piovezan et al. reported significantly reduced osteocalcin levels in PCOS women with BMI ≤ 27 kg/m^2^ (30). Additionally, it has been reported that lower serum levels of osteocalcin in PCOS subjects were related to androgen concentrations, IR, and polycystic appearance of the ovaries (31). However, this critical aspect of the PCOS-BMD relationship should be more thoroughly assessed.

Furthermore, and in contrast to many previous reports, the serum levels of vitamin D and PTH did not differ between the two groups in the current study. Still, phosphate levels were higher in PCOS patients independent of vitamin D and PTH values. Recent literature has suggested that elevated phosphorus levels, by their influences on IR, might play a role in developing PCOS (32-34). Nevertheless, higher phosphate levels in the present study’s PCOS cases were not correlated to testosterone, insulin concentration, and HOMA-IR. Dietary intake and renal excretion might have affected the serum levels of phosphorus in this study. However, impaired renal function was one of the exclusion criteria in the present study.

In the current study, there was also a negative correlation between estrogen and phosphate levels in the PCOS group. Limited reports concern the association between sex hormones and serum phosphate in PCOS patients. Of note, gonadal steroids have recently been considered phosphate regulators. Additionally, estradiol treatment has been shown to induce phosphaturia in women through a PTH-independent mechanism (35). To the best of our knowledge, the present study is the first to show a negative relationship between serum phosphorus and estrogen levels in PCOS subjects independent of age, BMI, and HOMA-IR. Therefore, it is supposed that higher phosphate levels in the present study’s PCOS cases could indirectly result from androgen excess and relative hypoestrogenemia in these patients. Another finding, which has not been reported previously, was that serum phosphate concentrations were positively correlated with circulating osteocalcin levels in PCOS patients, independent of BMI. However, it is necessary to conduct further studies in the future to approve or decline these associations and to clarify the possible etiologies.

However, according to the present study’s hypothesis, no difference was observed regarding bone density between PCOS patients and controls. In addition, a positive and independent association was shown between IR, BMI, and bone density in PCOS patients but only between BMI and bone parameters in controls. Therefore, it seems that both IR and obesity are significant predictors of bone density in PCOS subjects.

One of the strengths of this study was the measurement of several hormones and the adjustment for multiple possible confounding factors, which helped reduce biases in the obtained results. Additionally, bone indices were measured in multiple skeletal sites (i.e., distal radius and total hip in addition to LS and FN). However, the study had some limitations, including its cross-sectional study design, which limited the conclusions regarding causality, the lack of measurement of dietary phosphorus intake, and the small sample size, which could lead to inadequate power for determining significant differences regarding bone density between the two groups.

### 5.1. Conclusions

The present study’s findings demonstrated that PCOS status was not a predictor of BMD. In addition, BMI and HOMA-IR were significant determinants of bone mineral density in PCOS women; however, BMI was the only determinant in healthy women. Polycystic ovary syndrome patients had higher serum phosphate levels. Nevertheless, the two groups were comparable regarding PTH and vitamin D concentrations. Serum osteocalcin was inversely correlated with BMI in both groups. Moreover, higher phosphate levels in the studied PCOS cases were inversely correlated with estrogen levels.

Nevertheless, the current study’s findings necessitate further investigation to specify the relative influence of hormonal and metabolic alterations in PCOS on bone health. For instance, whether there is any difference between healthy women and those with androgen excess regarding bone quality. In addition, it is noteworthy to determine whether defective insulin signaling and decreasing osteocalcin levels might have detrimental effects on bone density in PCOS women.

## Data Availability

The dataset presented in the study is available on request from the corresponding author during submission or after publication.

## References

[1] Fauser BC, Tarlatzis BC, Rebar RW, Legro RS, Balen AH, Lobo R (2012). Consensus on women's health aspects of polycystic ovary syndrome (PCOS): the Amsterdam ESHRE/ASRM-Sponsored 3rd PCOS Consensus Workshop Group.. Fertil Steril..

[2] Randeva HS, Tan BK, Weickert MO, Lois K, Nestler JE, Sattar N (2012). Cardiometabolic aspects of the polycystic ovary syndrome.. Endocr Rev..

[3] Rotterdam ESHRE/ASRM-Sponsored PCOS Consensus Workshop Group (2004). Revised 2003 consensus on diagnostic criteria and long-term health risks related to polycystic ovary syndrome.. Fertil Steril..

[4] Notelovitz M (2002). Androgen effects on bone and muscle.. Fertil Steril..

[5] Clarke BL, Khosla S (2009). Androgens and bone.. Steroids..

[6] Barber TM, Hanson P, Weickert MO, Franks S (2019). Obesity and polycystic ovary syndrome: Implications for pathogenesis and novel management strategies.. Clin Med Insights Reprod Health..

[7] Barber TM, McCarthy MI, Wass JA, Franks S (2006). Obesity and polycystic ovary syndrome.. Clin Endocrinol (Oxf)..

[8] Yuksel O, Dokmetas HS, Topcu S, Erselcan T, Sencan M (2001). Relationship between bone mineral density and insulin resistance in polycystic ovary syndrome.. J Bone Miner Metab..

[9] Guntur AR, Rosen CJ (2012). Bone as an endocrine organ.. Endocr Pract..

[10] Karadag C, Yoldemir T, Gogas Yavuz D (2017). Determinants of low bone mineral density in premenopausal polycystic ovary syndrome patients.. Gynecol Endocrinol..

[11] Ganie MA, Chakraborty S, Sehgal A, Sreejith M, Kandasamy D, Jana M (2018). Bone mineral density is unaltered in women with polycystic ovary syndrome.. Horm Metab Res..

[12] Katulski K, Slawek S, Czyzyk A, Podfigurna-Stopa A, Paczkowska K, Ignaszak N (2014). Bone mineral density in women with polycystic ovary syndrome.. J Endocrinol Invest..

[13] Yang HY, Lee HS, Huang WT, Chen MJ, Chen SC, Hsu YH (2018). Increased risk of fractures in patients with polycystic ovary syndrome: a nationwide population-based retrospective cohort study.. J Bone Miner Metab..

[14] Ferriman D, Gallwey JD (1961). Clinical assessment of body hair growth in women.. J Clin Endocrinol Metab..

[15] Kohrt WM, Bloomfield SA, Little KD, Nelson ME, Yingling VR, American College of Sports M (2004). American college of sports medicine position stand: Physical activity and bone health.. Med Sci Sports Exerc..

[16] Zborowski JV, Cauley JA, Talbott EO, Guzick DS, Winters SJ (2000). Clinical Review 116: Bone mineral density, androgens, and the polycystic ovary: the complex and controversial issue of androgenic influence in female bone.. J Clin Endocrinol Metab..

[17] Zborowski JV, Talbott EO, Cauley JA (2001). Polycystic ovary syndrome, androgen excess, and the impact on bone.. Obstet Gynecol Clin North Am..

[18] Adami S, Zamberlan N, Castello R, Tosi F, Gatti D, Moghetti P (1998). Effect of hyperandrogenism and menstrual cycle abnormalities on bone mass and bone turnover in young women.. Clin Endocrinol (Oxf)..

[19] Buchanan JR, Myers C, Lloyd T, Leuenberger P, Demers LM (1988). Determinants of peak trabecular bone density in women: the role of androgens, estrogen, and exercise.. J Bone Miner Res..

[20] Kasperk C, Helmboldt A, Borcsok I, Heuthe S, Cloos O, Niethard F (1997). Skeletal site-dependent expression of the androgen receptor in human osteoblastic cell populations.. Calcif Tissue Int..

[21] Carmina E, Guastella E, Longo RA, Rini GB, Lobo RA (2009). Correlates of increased lean muscle mass in women with polycystic ovary syndrome.. Eur J Endocrinol..

[22] Salamat MR, Salamat AH, Janghorbani M (2016). Association between obesity and bone mineral density by gender and menopausal status.. Endocrinol Metab (Seoul)..

[23] Wijeyaratne CN, Dilini Udayangani SA, Balen AH (2013). Ethnic-specific polycystic ovary syndrome: epidemiology, significance and implications.. Expert Rev Endocrinol Metab..

[24] Khan MJ, Ullah A, Basit S (2019). Genetic basis of polycystic ovary syndrome (PCOS): Current perspectives.. Appl Clin Genet..

[25] Dagogo-Jack S, al-Ali N, Qurttom M (1997). Augmentation of bone mineral density in hirsute women.. J Clin Endocrinol Metab..

[26] Clemens TL, Karsenty G (2011). The osteoblast: an insulin target cell controlling glucose homeostasis.. J Bone Miner Res..

[27] Srikanthan P, Crandall CJ, Miller-Martinez D, Seeman TE, Greendale GA, Binkley N (2014). Insulin resistance and bone strength: findings from the study of midlife in the United States.. J Bone Miner Res..

[28] Bezerra dos Santos Magalhaes K, Magalhaes MM, Diniz ET, Lucena CS, Griz L, Bandeira F (2013). Metabolic syndrome and central fat distribution are related to lower serum osteocalcin concentrations.. Ann Nutr Metab..

[29] Ferron M, Wei J, Yoshizawa T, Del Fattore A, DePinho RA, Teti A (2010). Insulin signaling in osteoblasts integrates bone remodeling and energy metabolism.. Cell..

[30] Piovezan JM, Premaor MO, Comim FV (2019). Negative impact of polycystic ovary syndrome on bone health: a systematic review and meta-analysis.. Hum Reprod Update..

[31] Diamanti-Kandarakis E, Livadas S, Katsikis I, Piperi C, Mantziou A, Papavassiliou AG (2011). Serum concentrations of carboxylated osteocalcin are increased and associated with several components of the polycystic ovarian syndrome.. J Bone Miner Metab..

[32] Mahmoudi T, Gourabi H, Ashrafi M, Yazdi RS, Ezabadi Z (2010). Calciotropic hormones, insulin resistance, and the polycystic ovary syndrome.. Fertil Steril..

[33] Chang E, Donkin SS, Teegarden D (2009). Parathyroid hormone suppresses insulin signaling in adipocytes.. Mol Cell Endocrinol..

[34] Wu X, Zhang S, He H, Wang Y, Li J, Gao J (2022). Associations of serum phosphorus concentrations with insulin resistance and hyperandrogenemia in normal weight women with polycystic ovary syndrome.. Research Square..

[35] Faroqui S, Levi M, Soleimani M, Amlal H (2008). Estrogen downregulates the proximal tubule type IIa sodium phosphate cotransporter causing phosphate wasting and hypophosphatemia.. Kidney Int..

